# Cystic Echinococcosis: Clinical, Immunological, and Biomolecular Evaluation of Patients from Sardinia (Italy)

**DOI:** 10.3390/pathogens9110907

**Published:** 2020-10-30

**Authors:** Cinzia Santucciu, Piero Bonelli, Angela Peruzzu, Alessandro Fancellu, Vincenzo Marras, Antonello Carta, Scilla Mastrandrea, Giorgio Bagella, Toni Piseddu, Stefano Profili, Alberto Porcu, Giovanna Masala

**Affiliations:** 1 OIE Reference Laboratory for Echinococcosis, National Reference Center for Echinococcosis (CeNRE), IZS della Sardegna, 07100 Sassari, Italy; piero.bonelli@izs-sardegna.it (P.B.); angela.peruzzu@izs-sardegna.it (A.P.); scilla.mastrandrea@izs-sardegna.it (S.M.); toni.piseddu@izs-sardegna.it (T.P.); giovanna.masala@izs-sardegna.it (G.M.); 2 Department of Medical, Surgical and Experimental Sciences, University of Sassari, 07100 Sassari, Italy; afancel@uniss.it (A.F.); alberto@uniss.it (A.P.); 3 Department of Biomedical Sciences, Institute of Pathology, University of Sassari, 07100 Sassari, Italy; marrasv@gmail.com; 4 U.O.C. di Radiologia, A.O.U., 07100 Sassari, Italy; Antonio.carta@aousassari.it (A.C.); sprofili@aslsassari.it (S.P.); 5 U.O.C. di Malattie Infettive, A.O.U., 07100 Sassari, Italy; 6 Radiology Unit, University Hospital of Sassari, 07100 Sassari, Italy; giorgio.bagella@tiscali.it

**Keywords:** *Echinococcus granulosus*, cystic echinococcosis, hydatid disease, human cystic echinococcosis, diagnosis of echinococcosis

## Abstract

Cystic echinococcosis (CE), a zoonotic disease caused by the larval stage of the tapeworm *Echinococcus granulosus sensu lato* (*s.l.*), is a worldwide public health problem. *Echinococcus granulosus sensu stricto* (*s.s.*), associated with G1 and G3 genotypes, is endemic with high prevalence in the Mediterranean basin. The parasite’s life cycle comprises definitive hosts (canids) and intermediate hosts (ruminants) and can occasionally involve humans. The main aim of this research was to confirm the diagnosis of 13 patients suspected of CE who presented different complications and needed the surgical removal of the cysts. We also wanted to understand and clarify more the diagnosis of echinococcosis in humans. For this purpose, the patients first underwent cyst evaluation by ultrasound (US), immunological analysis, and then total pericystectomy, followed by parasitological, histopathological, and molecular biology examinations of the cysts. US stadiated one CE1, one CE2, eight CE3b, one CE4, and two CE5; immunology evidenced nine positives; histopathology confirmed 11 CE cysts, of which 8 fertile presenting protoscoleces were identified as *E. granulosus s.s.* by molecular biology, genotyped as three G1 and four G3 by neighbor-joining (NJ) phylogenetic tree. In conclusion, the results showed that 11 patients were affected by *E. granulosus s.s.* G1 orG3, and 2 cystic neoformations were of non-parasitic origin.

## 1. Introduction

Human echinococcosis is a zoonotic disease caused by a species of medical importance, the *Echinococcus granulosus sensu lato* (*s.l.*), an etiological agent of cystic echinococcosis (CE) [1,2].

Human CE is an important public health problem with worldwide distribution, since it has been reported in all continents except Antarctica [3,4,5]. The areas in which the burden of infection is particularly relevant are South America, North Africa, Central Asia, Eastern Europe, Russia, the Near and Middle East, Western China, and the Mediterranean littoral [6,7].

Italy has an annual CE incidence of 1.6/10^5^ inhabitants. Human CE is distributed in all the territories, with several regional variations (sporadic, endemic, and hyperendemic). Sardinia and Sicily present the highest CE average incidence, and the observed rates correspond to 6.8/10^5^ and 4.0/10^5^, respectively [8]. Moreover, based on a recent report on the Hospital Discharge Records (HDRs) from 2001 to 2014, the Italian average incidence rates are linked to a cost of more than 53 million euros and about 3000 disability-adjusted life years (DALYs), being the highest annual average of costs and DALYs observed in Sardinia and Sicily [9].

The infection is closely associated with pastoral communities characterized by low standards of hygienic conditions [10], where dogs and livestock are raised together [11,12] close to humans. In particular, clandestine home slaughter, mainly of goats and/or sheep and associated with the presence of sheepdogs and stray dogs, enable the spread of CE in the Mediterranean basin [6]. According to the World Health Organization (WHO) [13], CE has been described as one of the 17 neglected tropical diseases, and it has been listed along with the most severe parasitic diseases in humans; moreover, it has been ranked second among the food-borne animal parasitoses. In addition, at the first Joint Expert Committee on Zoonoses, held in 1950, CE was evaluated as an important public health problem all over the world and an economic issue [14,15]. Furthermore, CE has been estimated to be the cause of the yearly loss of more than 1 million disability-adjusted life years (DALYs) [2].

The etiological agent of CE is the larval stage of *Echinococcus granulosus* (*s.l.*), which belongs to the family of Taeniidae [16,17]. 

*E. granulosus s.l.* presents a large variability in infectivity to humans, pathogenicity, developing rate, antigenicity, host range, morphology, and genetic diversity [16,17,18,19,20]. 

Various species and genotypes of *E. granulosus s.l.* have been already identified by molecular characterization studies [21,22,23,24,25,26,27]. 

Based on more recent studies on nuclear and mitochondrial genes, *E. granulosus* complex has been grouped into five diverse species, which are taxonomically classified as follows: *E. granulosus sensu stricto* (*s.s.*) (genotypes G1 and G3), *E. equinus* (G4), *E. ortleppi* (G5), *E. canadensis* (G6–G8 and G10), and *E. felidis* [17,23,25]. These species are epidemiologically and geographically distinct. In particular, *E. granulosus s.s.* is endemic and presents a high diffusion in all of the Mediterranean basin, in which this genotype is the main causative agent of CE in humans [4,16]. 

The parasite life cycle of *Echinococcus* species first comprises a definitive host, such as canids, that may hold the adult worm in the intestine, which could release eggs into the environment through the feces. The next step is an intermediate host, typically represented by ruminants, and only atypically by humans, since human infection has no role in maintaining the lifecycles and represents an epidemiological dead end [3,28,29,30,31]. For these reasons, humans are considered accidental and aberrant hosts, as are other carnivores [32]. The intermediate host may ingest food contaminated with eggs; later, in the larval form known as metacestode, they can give rise to a fluid-filled cyst in diverse organs, mainly the liver (70% to 85% of cases) and/or the lungs, (20% of cases) and may remain asymptomatic for years [33,34,35]. Less than 10% are found in other sites, including bone, brain, and spleen [3]. Daughter cysts can also develop inside them [36,37].

The WHO Informal Working Group on Echinococcosis (WHO-IWGE) set up a classification of hydatids, based on specific stages, that allows classification of the cysts into the following three relevant groups: (1) two substages of the “active” group include developing cysts, which may be unilocular (CE1) or multi-vesicular with daughter vesicles (CE2) and which are usually found to be viable; (2) the “transitional” group (CE3), which includes both cysts with detachment of endocyst (CE3a) and predominantly solid cysts with daughter vesicles (CE3b); and (3) the “inactive” group (CE4 and CE5), which exhibit involution and solidification of cyst content with increasing degrees of calcification and are nearly always found to be non-viable [2,38,39,40]. This classification helps with managing the patient and with diagnosis, treatment, and follow-up. 

The stadiation of the cyst is mostly performed by means of imaging techniques, which are indispensable tools for diagnosing CE. These methods comprise ultrasound (US), magnetic resonance imaging (MRI), computed tomography (CT), and/or conventional chest radiography. These exams make it possible to establish not only the specific stage of the hydatids but also the localization. Whenever possible, MRI should be preferred to CT due to better visualization of liquid areas within the matrix [39,41]. In contrast, differential diagnosis involves infectious lesions and tumors [3].

To support the findings of imaging techniques for CE diagnosis and follow-up, serological analysis represents a useful tool and has been regularly used as a screening or confirmatory test [42]. 

Currently, the main immunological methods for CE patients for diagnosis and follow-up are enzyme-linked immunosorbent assays (ELISAs), used as a screening test, and immunoblotting (IB), employed as a confirmatory assay, since IB has higher specificity and sensitivity than other techniques [42]. Other tests, such as indirect hemagglutination assay (IHA), immunochromatographic test (ICT), immunofluorescence assay (IFA), and dot immunogold filtration assay (DIGFA), often present lower sensitivity and specificity and are consequently employed less. All these immunological tests aim to detect specific IgG antibodies anti-*E. granulosus* [42,43,44,45,46,47,48]. 

Radiological techniques and immunological diagnosis, when possible, are supported by molecular diagnosis of DNA-based analysis. It is a very useful tool, since it gives a wider and complete diagnostic picture of CE patients. Moreover, the polymerase chain reaction (PCR) technique presents high specificity and sensitivity, and it is likewise very helpful to confirm the diagnosis, as well as for the identification of the genus, species, and genotype [42]. 

The main aim of this research was to confirm the diagnosis of 13 patients suspected of CE. We also wanted to understand and clarify more the diagnosis of echinococcosis in human patients. For this purpose, the patients first underwent immunological analysis and cyst evaluation by imaging techniques. Then, several examinations were performed on the cysts after the total pericystectomy, such as parasitological cyst inspection and histopathological and molecular biology analysis. The results obtained were analyzed, evaluated, and compared to assess their agreement or discrepancy. However, among two tests used for sera analysis of CE patients, existing guidelines necessitate at least one positive serological result to define a case as confirmed or probable, and immunology has to be associated with epidemiological, clinical, imaging, and parasitological evidence [38,39].

## 2. Results

### 2.1. Radiological Examination 

Radiological findings for the 13 patients investigated revealed in each subject the presence of one or more hepatic neoformations attributable to echinococcal cysts for the typical features. The cysts ranged from 3 cm to 20 cm in diameter and were variable among patients. 

Moreover, according to our results displayed in Table 1, stadiation of the 13 patients’ hydatids revealed that one cyst was CE1 (HCE1), one was CE2 (HCE2), no CE3a was detected, eight hydatids were CE3b (from HCE3 to HCE9 and HCE12), one corresponded to CE4 (HCE10), and three belonged to CE5 (from HCE11 to HCE 13). For the HCE4 the patient, who harbored three cysts, classification was clear only for a CE3b. On the other hand, HCE10 and HCE12 had two formations both corresponding to CE3b and CE5. However, for the latter, only the CE5 was delivered to the laboratory for further analysis.

### 2.2. Serology Analysis

The serology results for the 13 sera examined (Table 1) by the screening method using the “*Echinococcus* IgG” ELISA kit (DRG Instruments GmbH, Marburg, Germany) showed eight positive and five negative samples. In contrast, the analysis performed using the confirmatory test IB-*Echinococcus* Western Blot IgG (LDBIO-Diagnostics, Lyons, France) detected nine positive and four negative sera.

### 2.3. Surgery

The presence, localization, dimension, and number of the cysts were confirmed at the time of the surgery, in accordance with the previously reported radiological findings, in the group of 13 patients after the total pericystectomy.

**Figure 1:**
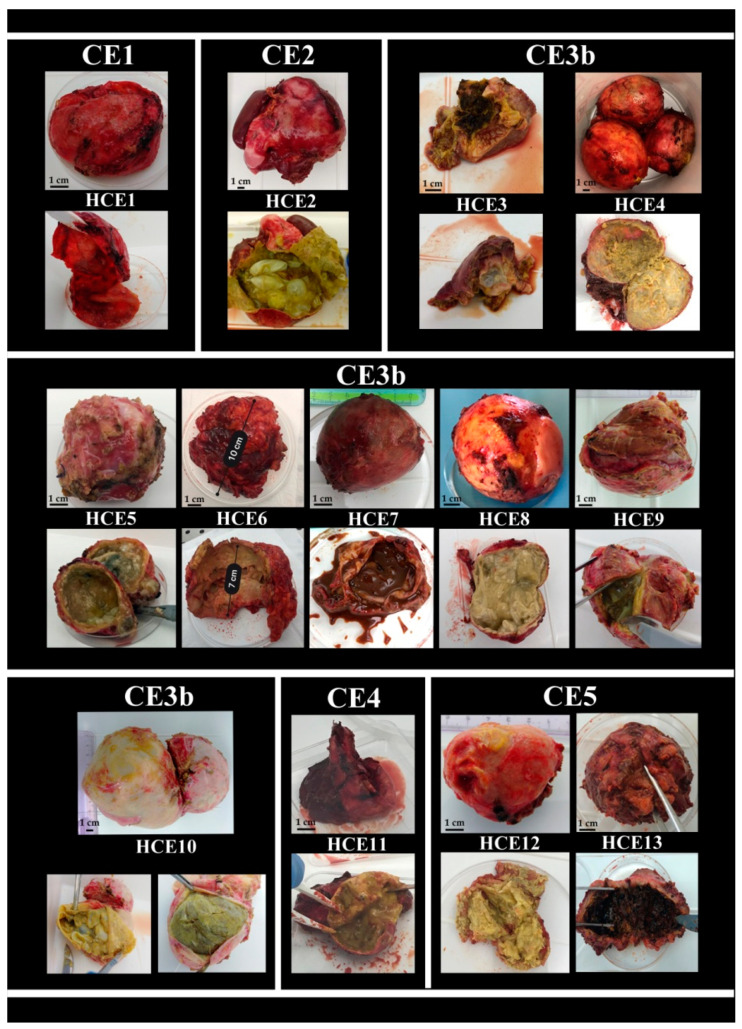
External and internal features of cysts surgically enucleated from 13 patients, displayed according to ultrasound (US) stadiation.

From the liver of each patient, one or more cysts, from HCE1 to HCE13, were enucleated. The neoformations presented a wide variety of characteristics, already evidenced by US, in diameter (ranging from 6 cm to 20 cm), shape (elliptical or spherical), consistency (soft or solid), and number, as displayed in Figure 1 and confirmed also by parasitological examination.

One patient receiving emergency surgery to remove a recurrent hydatid cyst measuring 7 × 8 cm in contact with the hepatic hilum and complicated by abscessualization and septic shock, deceased from hemorrhagic shock on postoperative day 6.

The 13 patients underwent a regular post-surgical follow-up, which consisted of monitoring them by US.

### 2.4. Hydatid Cyst Examination

The hydatid cyst examination of both the external and the internal portions evidenced several differences between the neoformations, as shown in Figure 1. In the panel, the images of all the cysts surgically removed (HCE1-> HCE13) are displayed according to their radiological stadiation (as reported above: one CE1, one CE2, eight CE3b, one CE4, and two CE5). Differences between the cysts are evidenced for both the external and the internal parts. Concerning the internal content of HCE1, no evaluation was possible for its absence, as already reported. Regarding HCE7, the internal material was chocolate brown colored, smelly and liquid, and could be aspirated by a syringe. No membranes or daughter cysts were visible. On the other hand, the inner wall of HCE13 differed completely from those of the other cysts since it presented as a whole clot. 

The analysis of the inner biological material by the microscope evidenced the presence of protoscoleces, or parts of them such as hooks, in eight samples (Table 2), proving that these cysts were fertile. However, no related movement was detected; consequently, protoscoleces were considered non-viable. 

**Figure 2:**
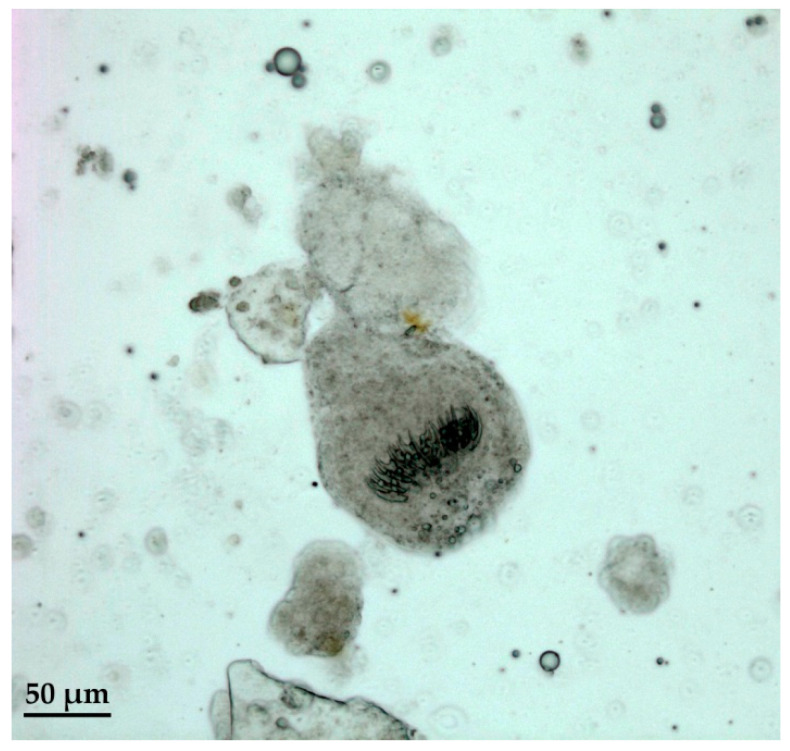
Protoscoleces with visible hooks.

A detail of protoscoleces observed by microscope is shown in Figure 2.

### 2.5. Histopathological Examination

The analysis of the slides prepared for the histopathological examination for the 13 samples (HCE1-HCE13) collected during the surgical procedure evidenced characteristics that fit with those of a parasitic cyst for 11 out of the 13 samples examined. All these hydatids were confirmed as belonging to a parasite that fit completely with that of *E. granulosus* (Table 1). In contrast, the HCE7 and HCE13 were considered cystic neoformations of non-parasitic origin. 

**Figure 3:**
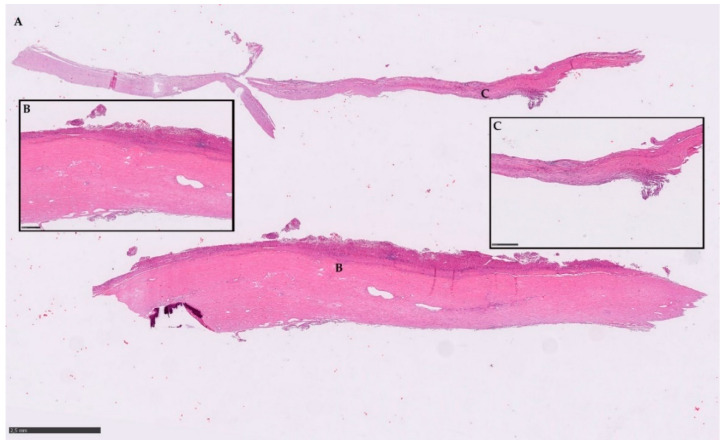
Histopathology section (**A**) representative of the typical parasitic material detected by microscope. (**B**) and (**C**) magnification of the correlated spot. Scale bars: 2.5 mm (**A**); insert frames 500 µm (**B**,**C**).

In particular, the histological picture of these 11 hydatid cysts evidenced different features, from the presence of numerous protoscoleces, within or outside the brood capsule in the fertile cysts, to a complete absence of protoscoleces or their parts. Moreover, the cyst wall was surrounded by the adventitial layer, which in turn was surrounded by the inflammatory tissue. A small part of the adjacent liver parenchyma was rarely present. In several cases, the cyst wall was calcified and/or sclerotic. A detail of a histopathology section is displayed in Figure 3.

### 2.6. DNA Amplification and Sequencing

Agarose gel electrophoresis evidenced the genomic DNAs of 8 out of the 13 samples examined. 

The quantifications of the eight genomic DNA samples, performed by a NanoPhotometer^®^ N120 (Implen GmbH, Munich, Germany), reported the following amounts of DNA: HCE2, 20.1 ng/µL; HCE3, 100.1 ng/µL; HCE4, 7.4 ng/µL; HCE5, 9.7 ng/µL; HCE6, 7.9 ng/µL; HCE8, 9.3 ng/µL; HCE9, 73.3 ng/µL, HCE10, 7.2 ng/µL (small cyst), and 345.5 ng/µL (big cyst).

Since the PCR for *E. granulosus s.s.* (PCR *E.g.s.s.*) amplified a sequences of 1001 bp of the Calreticulin (*Cal*) gene of eight DNA samples, they were identified and confirmed as *E. granulosus s.s*. G1 or G3 (Table 3). 

A fragment of the mitochondrial *COX1* gene was successfully amplified and sequenced from the parasite material of seven hydatid cysts collected in this study. DNA consensus sequences of 880 bp were obtained by trimming low-quality chromatogram data (Table 3). Unfortunately, the HCE4 sequence was of such poor quality that it was difficult to identify the genotype.

**Figure 4:**
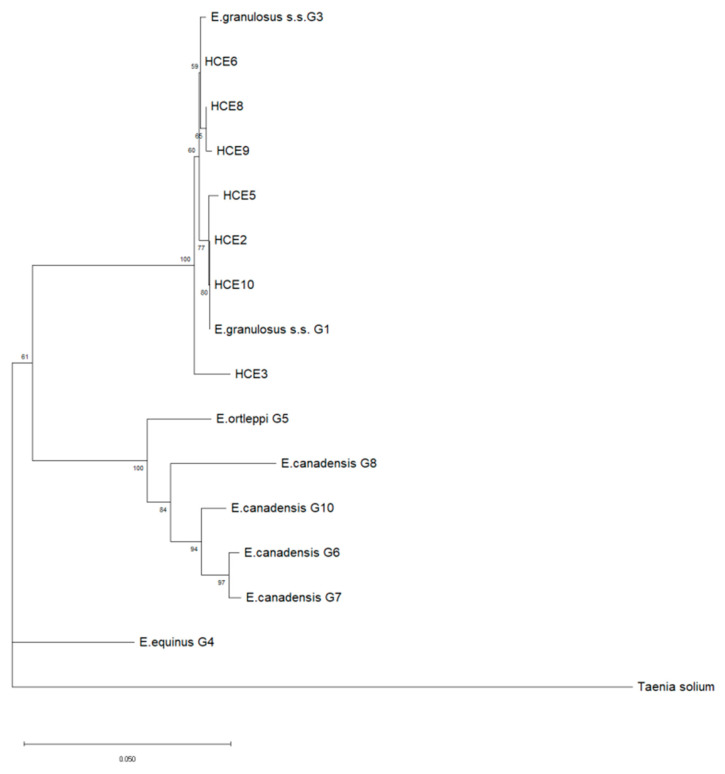
Phylogenetic tree of *Echinococcus granulosus sensu lato* (*s.l.*) samples collected from human patients affected by cystic echinococcosis (CE) (*n* = 7) based on a 720-bp-long portion of the mitochondrial *COX1* gene. MEGA X was used to construct a NJ tree based on the Kimura 2-parameter method. The reference sequences (*n* = 9) of *E. granulosus s.l.* were retrieved from the GenBank database. The reliability of the tree was assessed by 1000 bootstrap replications. Bar: number of base substitutions per site. Bootstrap values below 80% are not shown. A *Taenia solium* sequence was used as an outgroup.

The neighbor-joining (NJ) phylogenetic tree allowed the typing of human isolates as depicted in Figure 4. All of them were identified as *E. granulosus s.s.*, of which three belonged to G1 (HCE2, HCE5, HCE10) and four to G3 (HCE3, HCE6, HCE8, HCE9) genotypes (Table 3).

## 3. Discussion

This report is based on a multidisciplinary approach to the diagnosis of CE on human patients. When possible, the employment of different techniques is the unique key to guaranteeing a successful and correct diagnosis of this zoonosis.

CE diagnosis is still challenging, and many cases are asymptomatic for years, due to the absence of pathognomonic signs. CE is often diagnosed as an occasional finding; however, the most typical associable symptoms observed on clinical examination in patients are pain (82.3%), feeling of fullness or upper abdominal discomfort (65.1%), nausea (54.9%), mechanical jaundice (35.8%), and hepatomegaly (74.6%) [46,49]. Hence, CE is often underdiagnosed or detected only incidentally or when some complications arise. 

About 30% of cases, concerning patients with liver hydatidosis, present complications, such as anaphylaxis, cyst suppuration, adjacent organ and/or vase compression, and rupture [33,50,51]. Our data from the 13 patients confirmed the main causes for cyst exportation reported by other studies. In a few circumstances, complications of hydatid cysts may be life threatening and necessitate surgical intervention in urgent or emergency situations. Complications can occur for either infection of the cyst fluid or rupture of the cyst, such as abscess formation of the cyst rupture of the cysts in the biliary tree or peritoneal cavity, or even liver cysts with thoracic involvement. Surgery may be performed following different procedures that may be adopted according to the features of the cyst and may present different ranges of invasivity [52]. 

This study is based on the diagnostic evaluation of 13 patients that presented different complications and needed the surgical removal of the cysts by total pericystectomy [52]. The choice of the diagnostic method depends on the phase of the infection and the cyst stadiation [53,54].

However, out of the 13 patients involved in the study, only seven (HCE2, HCE3, HCE4, HCE5, HCE6, HCE9, and HCE10) presented a clear diagnostic picture, since their results were positive for all the tests carried out. Hence, these patients can undoubtedly be diagnosed as affected by echinococcosis, in particular, *E. granulosus s.s.*, genotype G1 or G3, which was defined as the etiological causative agent for each patient. Nevertheless, several inconsistencies were, instead, detected among the results obtained from the remaining six patients (HCE1, HCE7, HCE8, HCE11, HCE12, and HCE13) investigated. 

HCE1 results were positive by imaging techniques and it was stadiated as CE1; however, the serology was negative. A reasonable explanation could be given considering that hydatids at early stages do not usually develop an antibody response with a detectable titer [39,55,56]. As confirmed by other authors [39,40,42], false-negative results could occur in cases of CE in the liver with young CE1 cysts in a medium percentage (30–58%). Moreover, it may depend on different factors described in several studies, such as inactive (CE4 and CE5) cyst stages, single and small cysts, and cyst location other than the liver [47,48]. Moreover, it was not possible to perform the molecular biology in these samples, since they were delivered to the laboratory without the inner parasitic material. The diagnosis was finally confirmed by the histopathology that evidenced the typical structure attributable to a parasitic cyst, likely *E. granulosus*. 

HCE7 was stadiated by US as CE3b, but all tests performed were negative. The external features of this cyst were also compatible with a diagnostic frame of hydatid characteristics; however, the internal material was chocolate brown colored and smelly. The histopathology was not in contrast with a parasitic cyst. Hence, all results led to an inconclusive diagnosis. Further investigation should be necessary to evaluate the possibility of a hepatic abscess, which can often lead to a suspicion of echinococcal cyst in the early evaluation of the patient [57].

HCE8 presented positive results for all tests carried out, which were also confirmed by histopathology and molecular biology, and was associated with *E. granulosus s.s.* G3. However, the serological analysis of the routine ELISA was negative, contrary to the results of IB, which was employed as a confirmatory test. Serologic tests are very useful tools for confirming imaging techniques. Nevertheless, serodiagnosis in CE presents several limitations, not only for false-positive results [39,40], as cross-reaction with other parasitic and nonparasitic diseases can also occur [41,42,44,47] and must be considered to correctly interpret results. The employment of the right immunological test is important for detecting antibodies for CE diagnosis. Both ELISA and IHA are usually the first-line tests for CE patients, since they are rapid tests, prevent the wasting of time, and present a good sensitivity and specificity. Conversely, IB is used as a confirmatory test due to its high sensitivity and specificity, but it requires more time for the execution [42,44,47].

In addition, serological false-negative results (50–87%) have also been reported in cases of hepatic CE with old and inactive hydatids CE4 and CE5 [39,40,42], as well as for HCE11 stadiated CE4. The molecular biology also showed negative results. However, the histopathology described the typical structure attributable to a parasitic cyst, likely *E. granulosus*.

The cyst HCE12 analyzed in our laboratory presented a US stadiation corresponding to CE5. We expected a negative serology, as was the case for the molecular biology results. However, this patient also harbored a CE3b cyst that usually developed a positive immune response, which was in line with other reports on patients with multiple generally sero-positive cysts [40,48,58]. 

A multidisciplinary approach was essential to accomplish the diagnosis related to the patient linked to the HCE13 and clinically suspected of CE. In fact, the internal walls appeared entirely as clots (Figure 1), a feature completely different from that of an echinococcal cyst. The neoformation was evaluated by US as CE5, since the characteristics revealed a solid cyst with high degrees of calcification; nevertheless, both serology and molecular biology were negative. Finally, histopathology also gave a negative response for CE, and this neoformation was classified as a tumor. This case falls within those of differential diagnosis involving infectious lesions, abscesses, and tumors [3,57], which is one of the main problems related to the diagnostic field of CE.

A multimodal approach to the diagnosis of CE in humans takes advantage of a combination of different techniques so that the best approach for the clinical management of the patients may be chosen. The WHO’s expert consensus (2010) [38] reported indications to decide the best clinical management option based on a stage-specific approach in the case of hepatic localization of uncomplicated cysts, stadiated by US (as reference method) or magnetic resonance imaging. According to these guidelines, there are four treatment options for hydatidosis. For active echinococcal cysts, a pharmacological treatment (benzimidazole-class drugs) or its combination with the puncture-aspiration-injection-reaspiration (PAIR) technique is preferred; conversely, for inactive cysts of the liver, the watch-and-wait approach is best [53]. While surgery was once the most used treatment modality, it is currently reserved for rare selected cases such as complicated cysts, or complex cases in which minimally invasive management failed or was not feasible. The 13 cases included in the present study, despite the pharmacological treatment, underwent total pericystectomy for complex cases not suitable for either conservative or minimally invasive treatment. Although the ideal surgical treatment of liver hydatid cysts remains a matter of discussion, there is robust evidence that removal of the cysts along with the pericystium plays a pivotal role in preventing cyst recurrence. Interestingly, one of the patients of the present cohort received surgery for a recurrent cyst developing 10 years after a previous intervention, in which only evacuation of cystic material and deroofing of the cyst had been carried out, without removal of the pericystium.

However, this study presents several limitations, since it is only a small representative sample and provides a limited amount of information. Nonetheless, several incongruences detected among the different diagnostic tools employed, along with the two samples belonging to non-CE cysts, give some points of strength to this study, since they contribute to filling the gap on this still challenging CE diagnosis and its differentiation. We intend to increase the number of subjects in future studies, which could be helpful to understand more about human diagnosis of CE. Moreover, further studies of molecular biology will be performed on these 13 samples, such as on their genotype and haplotype to evaluate in more detail the species of *E. granulosus s.s.* from the island of Sardinia, Italy. 

## 4. Materials and Methods 

### 4.1. Study Design 

This study focused on a cohort of 13 patients belonging to a bigger group of about 100 subjects. Regrettably, these subjects presented significant complications; hence, they had to deal with the surgical removal of the cysts. All people involved underwent blood sampling for further immunological tests to detect antibodies against *E. granulosus,* along with diagnostic imaging that evidenced the patients presenting a neoformation with features attributable to CE. All serological results and the radiological information related to the hydatids (stadium, localization, and number) were evaluated along with all data of the surgical investigation. 

After the DNA extraction, the hydatid cysts surgically excided were examined for parasitological inspection, anatomopathological analysis, and biomolecular examinations for amplification and sequencing to identify the genotype. 

Data related to the cysts were used to confirm the diagnosis made by clinical evaluation. Finally, a phylogenetic tree was built using the human DNA isolates in our laboratories and other reference sequences of *E. granulosus s.l.* recovered from GenBank. 

### 4.2. Patients Involved in the Study 

A total number of 13 patients were enrolled in this study, including 7 females and 6 males aged 18 to 80 with an average age of 59.3 and a standard deviation of ±17.7 (Table 4). Three patients originated from other countries, namely China, Morocco, and Romania, but had been living in Italy for several years. These subjects were part of a bigger group of 105 people mainly of Italian nationality from the Sassari, Ozieri, and Nuoro Hospital Wards, located in Sardinia (Italy), who were investigated for CE from 2017 to 2020. 

Since these 13 patients (12.4%) presented complications (Table 5) associated with a remote pathologic anamnesis of echinococcosis, they had to undergo surgery for cyst excision. All patients had symptomatic hydatid cysts of the liver, despite the pharmacological anthelmintic therapy (albendazole) (GlaxoSmithKline Manufacturing S.p.A. Zentel, Verona, Italy).

All the patient’s medical procedures were performed according to the WHO-IWGE guidelines [39,40]. 

### 4.3. Ethical Statement

All procedures carried out in this research study, concerning the management of the patients and the human biological materials, were carried out in accordance with the rules of ethical standards of the Declaration of Helsinki of 1975, revised in 2013. 

Moreover, the ethics committee of the Local Health Authority of Sassari (Comitato di Bioetica, ASL N. 1, Sassari, Protocol n° 1136), approved the analyses of human samples by the Istituto Zooprofilattico Sperimentale of Sardinia according to the request of the National Health Service doctors, since 26 March 2013. Written informed consent was obtained from all patients or patients’ parents, depending on age.

### 4.4. Radiological Examination 

The 13 patients presenting complications first underwent image technique examination at the Radiologic Service of the Hospital Device of Sassari, Italy. The main exam was ultrasound (US). The instrument employed was the MyLabTwice (Esaote^®^, Genoa, Italy) with the probe convex (Mod.CA541, Esaote^®^) with a frequency of 6.5–7 MHz and linear with a variable band probe (Mod.LA523, Esaote^®^) with a frequency of 9–2 MHz. This tool could reveal any formation localized in the liver, only occasionally in other organs, such as the lungs if cysts were peripherally located, as reported elsewhere (El Fortia et al., 2006). 

When cysts were identified, their stadium was also ascertained (CE1, CE2, CE3a, CE3b, CE4, and CE5) by US according to diagnostic protocols of the WHO-IWGE [38,39]. Patients also underwent conventional radiography, which is useful to evidence a possible thoracic or bone involvement. Finally, computerized tomography (CT) and magnetic resonance (MR) imaging, as very sensitive tools, were employed, if necessary, to confirm the diagnosis or to have more information on localization, size, and number of cysts. 

### 4.5. Serology Analysis

Blood samples were taken from the 13 patients examined. Sterile tubes without any anticoagulant were employed, and if the clot was not completed, the tube was centrifuged at 1500× *g* for 5 min. 

Samples were analyzed by an immunological test employed for IgG antibodies detection in the diagnosis of *E. granulosus* and *E. multilocularis*, the ELISA “*Echinococcus* IgG” kit (DRG, Instruments GmbH, Marburg, Germany), habitually used in our laboratory for routine analysis, and then by the “*Echinococcus* Western Blot IgG” (IB) (LDBIO-Diagnostics, Lyons, France) regularly employed for the confirmatory diagnosis. The immunological tests were performed according to the manufacturer’s instructions.

### 4.6. Surgery

The 13 patients, hospitalized at the medical surgical ward, were submitted to surgery for a total pericystectomy, comprising the complete removal of the whole cyst and its fibrous capsule from the hepatic parenchyma [52]. These cysts were surgically removed from this group of patients, since they presented several complications due to particular clinical characteristics, such as large dimensions or a disadvantageous localization for compression of important blood vessels or bile duct and bacterial infection. 

After the surgery, the hydatids, completely enucleated, were put in a clean and dry sterile plastic container with a cap and then transported to our laboratory for further analysis. 

### 4.7. Hydatid Cyst Examination

Hydatid cysts, surgically isolated from the livers of 13 human patients (HCE1 to HCE13), were firstly analyzed by visual inspection to describe the external features. Afterward, to determine or confirm if cystic formations were caused by *E. granulosus*, the inner biological material from each hydatid was examined, with the exception of HCE1, since this sample was sent to the laboratory lacking its internal content. Three patients harbored multiple formations (HCE4, HCE10, and HCE12).

Following the resection of the outer capsule, the presence or absence of hydatid fluid and daughter cysts and/or membranes was ascertained by ocular analysis. 

Finally, the presence or absence of protoscoleces, or their parts, such as hooks, were also established with the support of a microscope. It was determined if the hydatid cyst was sterile or fertile. All the biologic materials were stored at −80 °C for further analyses. 

### 4.8. Histopathological Examination

After the previous examinations, the biological cystic materials (HCE1 to HCE13) collected during the surgical procedure from the livers of the 13 human patients were promptly fixed in 10% neutral formalin and then embedded in paraffin according to routine laboratory protocols for histopathological examination. Briefly, slides were prepared by cutting histological serially sections of the paraffin blocks at 4 µm and staining using hematoxylin and eosin.

### 4.9. DNA Extraction from Parasite Tissue 

Total genomic DNA was extracted from the parasite biological materials from each cyst belonging to the 13 human patients (HCE1-HCE13), detected following the isolation, respectively, of hydatid fluid and germinal layer. In detail, protoscoleces were removed by gently scraping the germinal layer placed in a Petri dish together with the hydatid fluid and then washed twice in phosphate-buffered saline (PBS) by a centrifuge step (10 min at 1000× *g*). Next, the supernatant was discarded, and 25 mg of the pellet were aliquoted and stored at −80 °C until the DNA extraction step. Conversely, negative control was obtained from a DNA sample extracted from a healthy human lymph node. DNA extraction was performed using the DNeasy Blood and Tissue Kit (Qiagen, Hilden, Germany) according to the manufacturer’s instructions. Total genomic DNAs were quantified by a NanoPhotometer^®^ N120 (Implen GmbH, Munich, Germany), according to the manufacturer’s instructions. Moreover, agarose gel electrophoresis was employed to check the presence and the integrity of the extracted total genomic DNAs. A 0.5% gel was prepared by melting the agarose powder in TAE buffer 1X; then, ethidium bromide (EtBr) was added to a final concentration of 0.5 μg/mL. DNA was loaded into the gel wells along with a molecular weight ladder. Finally, the electrophoretic run was performed at 150 V and 400 mA for about 1 hour.

### 4.10. DNA Amplification

Only DNA samples presenting the band related to the total genomic DNAs on electrophoresis were amplified by the following PCRs (Table 5):

- The PCR for *E.g.s.s.* [59] method was able to determine the species and the genotype of *E. granulosus s.s.* G1 or G3 in only one step, avoiding the sequencing step. This assay was performed by using the primers pairs that amplified the Cal gene of 1001 bp (F5′: CAATTTACGGTAAAGCAT-3′-R5′: CCTCATCTCCACTCTCT-3′) (Table 6). Primers were first prepared in a solution of 25 pmol/µL and 1.6 µM (1 pmol/µL final concentration), then 1 μL was used for the amplification of DNA along with 1 µL of DNA (3 ng/µL final concentration); in addition, 4 µL of Milli-Q water RNAse-free, 12.5 µL (1× final concentration) of 2x QuantiTect Probe PCR Master Mix (Qiagen), and 5.5 µL of Milli-Q water RNAse-free were added. The protocol consisted of an initial denaturation step at 95 °C for 15 min, followed by 35 cycles of 94 °C for 1 min, 56 °C for 30 s, and 72 °C for 1 min, and a final extension step at 72 °C of 5 min. After the end of the reaction, amplicons were stored at 4 °C before electrophoresis.
- PCR *COX1* [60] was employed to obtain an amplicon for the sequencing analysis of 880 bp, useful to differentiate between G1 and G3. The amplification of DNA was performed by using primers specific for the gene sequence of the enzyme cytochrome oxidase subunit I (*COX1*) (F 5′-TTTTTTGGCCATCCTGAGGTTTAT-3′ e R 5′-TAACGACATAACATAATGAAAATG-3′) (Table 6). Primers were first prepared in a solution of 25 pmol/µL and 1.6 µM (1 pmol/µL final concentration); then, 1 μL was used for the amplification of DNA, along with 1 µL of DNA (3 ng/µL final concentration), in addition to 4 µL of Milli-Q water RNAse-free, and 12.5 µL (1X final concentration) of 2x QuantiTect Probe PCR Master Mix (Qiagen). Finally, 5.5 µL of Milli-Q water RNAse-free were added. The protocol for the amplification was performed as follows: 1 cycle of 15 min at 95 °C, 40 cycles of 1 min at 94 °C, then 30 s at 58 °C, and 1 min at 72 °C, and 1 cycle of 5 min at 72 °C. After the end of the reaction, amplicons were stored at 4 °C before electrophoresis.

### 4.11. DNA Sequencing and Phylogenetic Analysis

After the purification of the amplicons by the *COX1* PCR, a sequencing analysis was performed with the Sanger method on their products using the QIAquick PCR Purification Kit (Qiagen). The total volume of reaction was 20 μL containing the following: 2 μL of BigDye Terminator 5X sequencing buffer, 4 μL of BigDye Terminator, 2 μL of H_2_O, 1 μL of Forward and Reverse primers, and finally 10 μL of DNA. The amplification protocol consisted of a total of 25 cycles, specifically, 10 s at 96 °C, 5 s at 57 °C, 2 min at 60 °C, and, in the last step, at 4 °C before the samples’ purification was performed by a chromatographic resin (Sephadex G-50 DNA grade F, Merck KGaA, Darmstadt, Germany). The sequencing step was carried out by an electrophoretic run into the automatic capillary sequencer 3500 (Genetic Analyzer, Applied Biosystems, Foster City, California, USA). 

A neighbor-joining phylogenetic tree was built on a dataset comprising the 7 human DNA isolates corresponding to HCE2 and HCE10, MK780827; HCE3, MK780842; HCE5, MK780830; HCE6, MK780839; HCE8, MK780843; and HCE9, MT991983; and other reference sequences of *E. granulosus s.l.*, such as *E. granulosus s.s.* G1: NC_044548; *E. granulosus s.s.* G3: KJ559023; *E. equinus* G4: AF346403; *E. ortleppi* G5: AB235846; *E. canadensis* G6: AB208063; *E. canadensis* G7: AB235847; *E. canadensis* G8: AB235848; *E. canadensis* G10: AB745463; and *Taenia solium*: AY211880 obtained from GenBank. 

## 5. Conclusions

The diagnosis of 13 patients suspected of CE showed 11 subjects infected by *E. granulosus s.s*. Only eight patients (HCE2, HCE3, HCE4, HCE5, HCE6, HCE8, HCE9, and HCE10) presented a clear diagnostic picture, since they were positive for all the tests carried out. These samples were genotyped as three G1 and four G3 by NJ phylogenetic tree. For the other five subjects investigated in the study, histopathology and/or molecular biology analysis of the cyst were of fundamental importance for a complete diagnostic picture. Three were confirmed to be affected by CE (HCE1, HCE11, and HCE12), whereas two patients (HCE7 and HCE13), with cystic neoformations of non-parasitic origin, were diagnosed as presenting a tumor and a hepatic abscess, respectively. 

These results confirm that imaging techniques and sero-diagnosis are the best tools employed only for in vivo diagnosis. However, in the present research, we corroborate that the multidisciplinary approach, if possible, is the best line to perform the diagnosis of CE on human patients. An accurate diagnosis allows for choosing the best clinical management for the patients and their follow-up.

## Figures and Tables

**Table 1:** Cyst stadium compared to serological findings.

Patients Cyst Id	Cyst Stadium	Serological Findings		
		ELISA (DRG) (OD) ^1^		IB (LDBIO) ^2^
HCE1	CE1	negative	/	negative
HCE2	CE2	positive	(2.6)	positive
HCE3	CE3b	positive	(3.6)	positive
HCE4	CE3b ^3^	positive	(0.7)	positive
HCE5	CE3b	positive	(1.2)	positive
HCE6	CE3b	positive	(2.3)	positive
HCE7	CE3b	negative	/	negative
HCE8	CE3b	negative	/	positive
HCE9	CE3b	positive	(2.2)	positive
HCE10	CE3b (CE5) ^4^	positive	(2.3)	positive
HCE11	CE4	negative	/	negative
HCE12	CE5 (CE3b) ^4^	positive	(2.5)	positive
HCE13	CE5	negative	/	negative

^1^ (OD): optical density, cut off: 0.6, ELISA-positive samples have an OD above the cut off; ^2^ Immunoblotting (IB)-positive samples presented a specific pattern that corresponded to the positive control; ^3^ 3 cysts (only stadium of one cyst was available); ^4^ 2 cysts.

**Table 2:** Presence or absences of protoscoleces.

Patients Cyst Id	Cyst Stadium	Protoscoleces
HCE1	CE1	Not available
HCE2	CE2	Non-viable
HCE3	CE3b	Non-viable
HCE4	CE3b	Non-viable
HCE5	CE3b	Non-viable
HCE6	CE3b	Non-viable
HCE7	CE3b	Negative
HCE8	CE3b	Non-viable
HCE9	CE3b	Non-viable
HCE10	CE3b	Non-viable
HCE11	CE4	Negative
HCE12	CE5	Negative
HCE13	CE5	Negative

**Table 3:** Results obtained by PCR for *Echinococcus granulosus sensu stricto* (*E.g.s.s.*), *COX1* genotyping, and sequencing of each cyst.

Cyst Id	Cyst Stadium	PCR for Gene *CAL* of 1001 bp	PCR for Gene *COX1* of 880 bp	Genotype	Sequence
HCE1	CE1	negative	negative	/	/
HCE2	CE2	positive	positive	G1	MK780827
HCE3	CE3b	positive	positive	G3	MK780842
HCE4	CE3b	positive	positive	/ *	/ *
HCE5	CE3b	positive	positive	G1	MK780830
HCE6	CE3b	positive	positive	G3	MK780839
HCE7	CE3b	negative	negative	/	/
HCE8	CE3b	positive	positive	G3	MK780843
HCE9	CE3b	positive	positive	G3	MT991983
HCE10	CE3b	positive	positive	G1	MK780827
HCE11	CE4	negative	negative	/	/
HCE12	CE5	negative	negative	/	/
HCE13	CE5	negative	negative	/	/

* the poor quality of the sequence made genotype and sequence identification difficult.

**Table 4:** Information related to the patients.

Patients Cyst Id	Gender	Age	Nationality
HCE1	Female	68	Italian
HCE2	Male	53	Italian
HCE3	Male	41	Chinese
HCE4	Male	75	Italian
HCE5	Female	75	Italian
HCE6	Male	43	Italian
HCE7	Female	67	Italian
HCE8	Female	60	Italian
HCE9	Male	64	Italian
HCE10	Female	42	Romanian
HCE11	Male	80	Italian
HCE12	Female	18	Moroccan
HCE13	Female	67	Italian

**Table 5:** Cyst stadium and list of complications related to each cyst.

Patients Cyst Id	Reason for Surgical Intervention
HCE1	Large viable cyst (6 × 7cm) in contact with hepatic veins
HCE2	Giant cyst (17 × 15 cm) with daughter cysts
HCE3	Recurrent large hydatid cyst (8 × 7 cm) complicated by abscessualization and septic shock
HCE4	Multiple large hydatid cysts (from 3 to 7 cm) with obstructive jaundice
HCE5	Large cyst (6 × 7 cm) causing recurrent cholangitis
HCE6	Large cyst (7 × 10 cm) ruptured into the biliary tree with jaundice and severe cholangitis
HCE7	Large viable cyst (7 × 6 cm) infiltrating the diaphragm and the chest wall
HCE8	Cyst (7 × 8 cm) in contact with the inferior vena cava causing recurrent cholangitis
HCE9	Large cyst (11 × 11 cm) abutting the diaphragm with biliary-bronchial fistula
HCE10	Multiple large cysts (from 7 to 10 cm) with recurrent cholangitis
HCE11	Large cyst (6 × 7 cm) with signs of abscessualization
HCE12	Multiple large cyst (6 × 7 cm), in contact with the portal vein, with daughter cysts and caseous
HCE13	Cyst (7 × 8 cm) fistulated into the biliary tree with recurrent cholangitis

**Table 6:** Description of different PCRs and primers employed.

Method	Gene Amplified	Primers	Length
PCR for *E.g.s.s.*	Calreticulin (*CAL*) l	F5′: CAA TTT ACG GTA AAG CAT-3′- R5′: CCT CAT CTC CAC TCTCT-3′	1001 bp
PCR *COX1*	Cytochrome oxidase subunit (*COX*) I	F 5′-TTGAATGCTTTGAGTGCTTG-3′ R 5′-GAACCTAACGACATAACATAATGA-3′	880 bp

## References

[1] Thompson R.C.A., Thompson R.C.A., Deplazes P., Lymbery A.J. (2017). Chapter Two—Biology and Systematics of Echinococcus. Advances in Parasitology.

[2] Agudelo Higuita N.I., Brunetti E., McCloskey C. (2016). Cystic echinococcosis. J. Clin. Microbiol..

[3] Eckert J., Gemmell M.A., Meslin F.-X., Pawłowski Z.S. (2001). WHO/OIE Manual on Echinococcosis in Humans and Animals: A Public Health Problem of Global Concern.

[4] Alvarez Rojas C.A., Romig T., Lightowlers M.W. (2014). Echinococcus granulosus sensu lato genotypes infecting humans—Review of current knowledge. Int. J. Parasitol..

[5] Marcinkute A., Šarkunas M., Moks E., Saarma U., Jokelainen P., Bagrade G., Laivacuma S., Strupas K., Sokolovas V., Deplazes P. (2015). Echinococcus infections in the Baltic region. Vet. Parasitol..

[6] Deplazes P., Rinaldi L., Alvarez Rojas C.A., Torgerson P.R., Harandi M.F., Romig T., Antolova D., Schurer J.M., Lahmar S., Cringoli G., Thompson R.C.A., Deplazes P., Lymbery A.J. (2017). Chapter Six—Global Distribution of Alveolar and Cystic Echinococcosis. Advances in Parasitology.

[7] Casulli A. (2020). Recognising the substantial burden of neglected pandemics cystic and alveolar echinococcosis. Lancet Glob. Health.

[8] Brundu D., Piseddu T., Stegel G., Masu G., Ledda S., Masala G. (2014). Retrospective study of human cystic echinococcosis in Italy based on the analysis of hospital discharge records between 2001 and 2012. Acta Trop..

[9] Piseddu T., Brundu D., Stegel G., Loi F., Rolesu S., Masu G., Ledda S., Masala G. (2017). The disease burden of human cystic echinococcosis based on HDRs from 2001 to 2014 in Italy. PLoS Negl. Trop. Dis..

[10] Craig P.S., Hegglin D., Lightowlers M.W., Torgerson P.R., Wang Q. (2017). Echinococcosis: Control and prevention. Adv. Parasitol..

[11] Siko S., Deplazes P., Ceica C., Tivadar C.S., Mogolin I., Popescu S., Cozma V. (2011). Echinococcus multilocularis in southeastern Europe Romania. Parasitology.

[12] Groeneveld F., Enstra A., Eding H., Toro M.A., Scherf B., Pilling D. (2010). Genetic diversity in farm animals a review. Anim. Genet..

[13] World Health Organization (2015). Investing to Overcome the Global Impact of Neglected Tropical Diseases. Third WHO Report on Neglected Tropical Diseases.

[14] Food and Agriculture Organization of the United Nations (FAO)/World Health Organization (WHO) (2014). Multicriteria-Based Ranking for Risk Management of Food-Borne Parasites. http://www.fao.org/publications/card/en/c/ee07c6ae-b86c-4d5f-915c-94c93ded7d9e/.

[15] Mableson H.E., Okello A., Picozzi K., Welburn S.C. (2014). Neglected zoonotic diseases-the long and winding road to advocacy. PLoS Negl. Trop. Dis..

[16] Romig T., Ebi D., Wassermann M. (2015). Taxonomy and molecular epidemiology of Echinococcus granulosus sensu lato. Vet. Parasitol..

[17] Nakao M., Yanagida T., Konyaev S., Lavikainen A., Odnokurtsev V.A., Zaikov V.A., Ito A. (2013). Mitochondrial phylogeny of the genus Echinococcus Cestoda: Taeniidae with emphasis on relationships among Echinococcus canadensis genotypes. Parasitology.

[18] Eckert J., Thompson R.C., Bucklar H., Bilger B., Deplazes P. (2001). Efficacy evaluation of epsiprantel Cestex against Echinococcus mutilocularis in dogs and cats. Berl. Munch. Tierarztl. Wochenschr..

[19] Thompson R.C. (2008). The taxonomy, phylogeny and transmission of Echinococcus. Exp. Parasitol..

[20] Gholami S., Irshadullah M., Mobedi I. (2011). Rostellar hook morphology of larval Echinococcus granulosus isolates from the Indian buffalo and Iranian sheep, cattle and camel. J. Helminthol..

[21] Bowles J., Blair D., McManus D.P. (1992). Genetic variants within the genus Echinococcus identified by mitochondrial DNA sequencing. Mol. Biochem. Parasitol..

[22] Bowles J., Blair D., McManus D.P. (1994). Molecular genetic characterization of the cervid strain ‘northern form’ of Echinococcus granulosus. Parasitology.

[23] Lavikainen A., Lehtinen M.J., Meri T., Hirvelä-Koski V., Meri S. (2003). Molecular genetic characterization of the Fennoscandian cervid strain, a new genotypic group G10 of Echinococcus granulosus. Parasitology.

[24] Nakao M., McManus D.P., Schantz P.M., Craig P.S., Ito A. (2007). A molecular phylogeny of the genus Echinococcus inferred from complete mitochondrial genomes. Parasitology.

[25] Huttner M., Nakao M., Wassermann T., Siefert L., Boomker J.D., Dinkel A., Sako Y., Mackenstedt U., Romig T., Ito A. (2008). Genetic characterization and phylogenetic position of Echinococcus felidis Cestoda: Taeniidae from the African lion. Int. J. Parasitol..

[26] Saarma U., Jõgisalu I., Moks E., Varcasia A., Lavikainen A., Oksanen A., Simsek S., Andresiuk V., Denegri G., González L.M. (2009). A novel phylogeny for the genus Echinococcus, based on nuclear data, challenges relationships based on mitochondrial evidence. Parasitology.

[27] Knapp J., Nakao M., Yanagida T., Okamoto M., Saarma U., Lavikainen A., Ito A. (2011). Phylogenetic relationships within Echinococcus and Taenia tapeworms Cestoda: Taeniidae: An inference from nuclear protein-coding genes. Mol. Phylogenet. Evol..

[28] Moks E., Jõgisalu I., Saarma U., Talvik H., Järvis T., Valdmann H. (2006). Helminthologic survey of the wolf Canis lupus in Estonia, with an emphasis on Echinococcus granulosus. J. Wildl. Dis..

[29] Moks E., Jõgisalu I., Valdmann H., Saarma U. (2008). First report of Echinococcus granulosus G8 in Eurasia and a reappraisal of the phylogenetic relationships of ‘genotypes’G5-G10. Parasitology.

[30] Deplazes P., van Knapen F., Schweiger A., Overgaauw P.A. (2011). Role of pet dogs and cats in the transmission of helminthic zoonoses in Europe, with a focus on echinococcosis and toxocarosis. Vet. Parasitol.

[31] Laurimae L., Davison J., Süld K., Plumer L., Oja R., Moks E., Keis M., Hindrikson M., Kinkar L., Laurimäe T. (2015). First report of highly pathogenic Echinococcus granulosus genotype G1 in dogs in a European urban environment. Parasit. Vectors.

[32] Bonelli P., Masu G., Dei Giudici S., Pintus D., Peruzzu A., Piseddu T., Santucciu C., Cossu A., Demurtas N., Masala G. (2018). Cystic echinococcosis in a domestic cat Felis catus in Italy. Parasite.

[33] Bhutani N., Kajal P. (2018). Hepatic echinococcosis: A review. Ann. Med. Surg..

[34] Chiboub H., Boutayeb F., Wahbi S., El Yacoubi M., Ouazzani N., Hermas M. (2001). Echinococcosis of the pelvic bone: Four cases. Rev. Chir. Orthop. Reparatrice Appar. Mot..

[35] Zhang W., Wen H., Li J., Lin R., McManus D.P. (2012). Immunology and immunodiagnosis of cystic echinococcosis: An update. Clin. Dev. Immunol..

[36] Díaz A., Casaravilla C., Irigoín F. (2011). Understanding the laminated layer of larval Echinococcus I: Structure. Trends Parasitol..

[37] Díaz A., Casaravilla C., Allen J.E. (2011). Understanding the laminated layer of larval Echinococcus II: Immunology. Trends Parasitol..

[38] Kern P., Menezes da Silva A., Akhan O., Müllhaupt B., Vizcaychipi K.A., Budke C., Vuitton D.A., Thompson R.C.A., Deplazes P., Lymbery A.J. (2017). Chapter Four—The Echinococcoses: Diagnosis, Clinical Management and Burden of Disease. Advances in Parasitology.

[39] Brunetti E., Kern P., Vuitton D.A. (2010). Expert consensus for the diagnosis and treatment of cystic and alveolar echinococcosis in humans. Acta Trop..

[40] Brunetti E., Tamarozzi F., Macpherson C., Filice C., Piontek M.S., Kabaalioglu A., Dong Y., Atkinson N., Richter J., Schreiber-Dietrich D. (2018). Ultrasound and Cystic Echinococcosis. Ultrasound Int. Open.

[41] Hosch W., Junghanss T., Stojkovic M., Brunetti E., Heye T., Kauffmann G.W., Hull W.E. (2008). Metabolic viability assessment of cystic echinococcosis using highfield 1H MRS of cyst contents. NMR Biomed..

[42] Siles-Lucas M., Casulli A., Conraths F.J., Müller N., Thompson R.C.A., Deplazes P., Lymbery A.J. (2017). Chapter Three—Laboratory diagnosis of Echinococcus spp. in human patients and infected animals. Advances in Parasitology.

[43] Zhang W., McManus D.P. (2006). Recent advances in the immunology and diagnosis of echinococcosis. FEMS Immunol. Med. Microbiol..

[44] Carmena D., Benito A., Eraso E. (2006). Antigens for the immunodiagnosis of Echinococcus granulosus infection: An update. Acta Trop..

[45] Barnes T.S., Deplazes P., Gottstein B., Jenkins D.J., Mathis A., Siles-Lucas M., Torgerson P.R., Ziadinov I., Heath D.D. (2012). Challenges for diagnosis and control of cystic hydatid disease. Acta Trop..

[46] Sarkari B., Rezaei Z. (2015). Immunodiagnosis of human hydatids disease: Where do we stand?. World J. Methodol..

[47] Manzano-Roman R., Sanchez-Ovejero C., Hernandez-Gonzales A., Casulli A., Siles-Lucas M. (2015). Serological diagnosis and follow-up of human cystic echinococcosis: A new hope for the future?. Biomed. Res. Int..

[48] Lissandrin R., Tamarozzi F., Piccoli L., Tinelli C., De Silvestri A., Mariconti M., Meroni V., Genco F., Brunetti E. (2016). Factors influencing the serological response in hepatic Echinococcus granulosus infection. Am. J. Trop. Med. Hyg..

[49] Patkowski W., Krasnodębski M., Grąt V., Masior Ł., Krawczyk M. (2017). Surgical treatment of hepatic Echinococcus granulosus. Gastroenterol. Rev..

[50] Yahya A.I., Shwereif H.E., Ekheil M.A., Ahmed S., Algader T.K.A., Gyaed F.O., Aldarat A.S. (2014). The role of emergency surgery in hydatid liver disease. World J. Emerg. Surg..

[51] Dinc T., Kayilioglu S.I., Akturk O.M., Coskun F. (2016). Surgical management of liver hydatid cyst related non-traumatic emergencies: Single center experience. Iran. J. Parasitol..

[52] Porcu A., Fancellu A., Cherchi G., Nigri G., Tsoulfas G., Hoballah J., Velmahos G., Ho Y.H. (2020). The role of emergency surgery in hydatid liver disease. The Surgical Management of Parasitic Diseases.

[53] Panteleev V., Nartaylakov M., Mustafin A., Abdeyev R., Salimgareyev I., Samorodov A., Musharapov D. (2019). Surgical treatment of liver echinococcosis and alveococcosis. Infez. Med..

[54] Lissandrin R., Tamarozzi F., Mariconti M., Manciulli T., Brunetti E., Vola A. (2018). Watch and Wait Approach for Inactive Echinococcal Cyst of the Liver: An Update. Am. J. Trop Med. Hyg..

[55] Pagnozzi D., Addis M.F., Biosa G., Roggio A.M., Tedde V., Mariconti M., Tamarozzi F., Meroni V., Masu G., Masala G. (2016). Diagnostic Accuracy of Antigen 5-Based ELISAs for Human Cystic Echinococcosis. PLoS Negl. Trop. Dis..

[56] Pagnozzi D., Tamarozzi F., Roggio A.M., Tedde V., Addis M.F., Pisanu S., Masu G., Santucciu C., Vola A., Casulli A. (2018). Structural and Immunodiagnostic Characterization of Synthetic Antigen B Subunits From Echinococcus granulosus and Their Evaluation as Target Antigens for Cyst Viability Assessment. Clin. Infect. Dis..

[57] Krige J.E.J., Beckingham I.J. (2001). Liver abscesses and hydatid disease. BMJ.

[58] Hernandez-Gonzalez A., Santivanez S., Garcia H.H., Rodriguez S., Munoz S., Ramos G., Orduna A. (2012). Improved serodiagnosis of cystic echinococcosis using the new recombinant 2B2t antigen. PLoS Negl. Trop. Dis..

[59] Santucciu C., Masu G., Mura A., Peruzzu A., Piseddu T., Bonelli P., Masala G. (2019). Validation of a one-step PCR assay for the molecular identification of Echinococcus granulosus sensu stricto G1-G3 genotype. Mol. Biol. Rep..

[60] Nakao M., Sako Y., Yokoama N., Fukunaga M., Ito A. (2000). Mitochondrial genetic code in cestodes. Mol. Biochem. Parasitol..

